# New insights on the biomineralisation process developing in human lungs around inhaled asbestos fibres

**DOI:** 10.1038/srep44862

**Published:** 2017-03-23

**Authors:** Fabrizio Bardelli, Giulia Veronesi, Silvana Capella, Donata Bellis, Laurent Charlet, Alessia Cedola, Elena Belluso

**Affiliations:** 1 CNR-Nanotec – Soft and Living matter Lab (S.Li.M. Lab) c/o Department of Physics, La Sapienza University, Piazzale Aldo Moro 5, 00185, Rome, Italy; 2 CNRS/CEA/University of Grenoble Alpes, Laboratoire Chimie et Biologie des Métaux (CBM-UMR 5249), 17, avenue des Martyrs, 38054, Grenoble, France; 3 European Synchrotron Radiation Facility (ESRF), 71, avenue des Martyrs, 38043, Grenoble, France; 4 Department of Earth Sciences, University of Torino, via Valperga Caluso 35, 10125, Torino, Italy; 5 Department of Pathological Anatomy, ASL-TO1, Martini Hospital, via Tofane 71, 10154, Torino, Italy; 6 Institute of Earth Science (ISTerre-OSUG UMR 5275), University of Grenoble Alpes, 1381, rue de la Piscine, 38400, Grenoble, France; 7 Centre for Studies on Asbestos and other Toxic Particulates “G. Scansetti”, University of Torino, via Pietro Giuria 9, 10125, Turin, Italy; 8 CNR IGG – Torino Unit, via Valperga Caluso 35, 10125, Torino, Italy

## Abstract

Once penetrated into the lungs of exposed people, asbestos induces an *in vivo* biomineralisation process that leads to the formation of a ferruginous coating embedding the fibres. The ensemble of the fibre and the coating is referred to as *asbestos body* and is believed to be responsible for the high toxicological outcome of asbestos. Lung tissue of two individuals subjected to prolonged occupational exposure to crocidolite asbestos was investigated using synchrotron radiation micro-probe tools. The distribution of K and of elements heavier than Fe (Zn, Cu, As, and Ba) in the asbestos bodies was observed for the first time. Elemental quantification, also reported for the first time, confirmed that the coating is highly enriched in Fe (~20% w/w), and x-ray absorption spectroscopy indicated that Fe is in the 3+ oxidation state and that it is present in the form of ferritin or hemosiderin. Comparison of the results obtained studying the asbestos bodies upon removing the biological tissue by chemical digestion and those embedded in histological sections, allowed unambiguously distinguishing the composition of the asbestos bodies, and understanding to what extent the digestion procedure altered their chemical composition. A speculative model is proposed to explain the observed distribution of Fe.

Occupational exposure to asbestos is universally associated with several lung injuries, including respiratory diseases, asbestosis, pleural mesothelioma, and, owing to other co-factors, lung cancer^1^,^2^. Asbestos fibres can enter living organisms by inhalation and manifest their toxicity after 20 to 40 years. For this reason, although asbestos started to be banned in most countries since the 1990s, a peak of mortality of hundreds thousands of victims is predicted for the next 5–10 years^3^,^4^,^5^. In addition, due to weathering of asbestos-reinforced cement products, environmental asbestos contamination is also becoming of concern among the general population, in particular in urban areas. These facts make asbestos a current major health threat worldwide. It is therefore of the utmost importance to carry on researches that can increase the awareness on this threat, push for more strict regulations, and help medical researchers finding more efficient treatments and prevention strategies.

Asbestos bodies (*AB*) are the product of a biomineralisation process resulting in the deposition of iron and organic matter (mainly proteins) around the inhaled asbestos fibres (both ferrous (amphiboles) and non-ferrous (serpentine, tremolite) asbestos^6^,^7^), and their count is one of the most accessible and established tools to assess the degree of exposure to asbestos for legal actions^6^. It was believed that the coating surrounding the fibres was a protective mechanism produced by macrophages in the attempt to segregate the cytotoxic fibres from the biological tissues^8^,^9^. More recently, other authors suggested that the coating material itself could enhance the cytotoxic properties of asbestos by favouring the generation of reactive oxygen species^10^,^11^. In agreement with these studies, iron on the surface of *AB* was demonstrated to be catalytically active^11^,^12^, and able to induce single strand breaks in DNA^13^. A pioneer study exploiting transmission electron microscopy (TEM)^7^, indicated that the fibres’ coating contains crystalline particles of the same order of size of the inorganic iron core of the ferritin molecule. On this basis, it was hypothesized that the crystalline material comprising the major part of the *AB* is composed of ferritin. It is nowadays widely accepted that the coating mainly consists of mucopolysaccharides and of an iron-storage protein (ferritin or hemosiderin). However, mainly due to technical limitations of laboratory analytical tools, the elemental composition and distribution, and the chemical form of iron in the *AB* is still not well established, preventing the formulation of solid hypotheses on the carcinogenesis. Synchrotron radiation micro-probe techniques^14^,^15^ are among the few tools with the resolution and sensitivity required to study the composition of micrometric *AB in situ* (i.e. embedded in the original biological tissue).

To better describe and understand the biomineralisation process occurring around asbestos fibres, asbestos-contaminated lung tissue from two former workers of an asbestos plant in North-West Italy was investigated. The plant produced fibre-reinforced cement products (90% cement, 10% asbestos), and, although it was dismissed in the mid-eighties, thousands of the workers, their relatives, and many inhabitants of the villages close to the plants, died, and are still dying, of asbestos-related diseases. An interdisciplinary and multi-technique approach was followed: two synchrotron radiation based micro-probe techniques, namely micro X-Ray Fluorescence (μXRF) and micro X-ray Absorption Spectroscopy (μXAS), were combined with Scanning Electron Microscopy (SEM) and Elemental Dispersive Spectroscopy (EDS), and the results were interpreted taking in consideration altogether the mineralogical, geochemical, and biological aspects of the topic. With respect to the previous works exploiting similar synchrotron based techniques for this topic^16^,^17^, this work focuses on elements heavier than Fe, and on the comparison between the composition of the *AB* isolated by the digestion of the biological tissue and those embedded in the original lung tissue.

## Results

**Figure 1:**
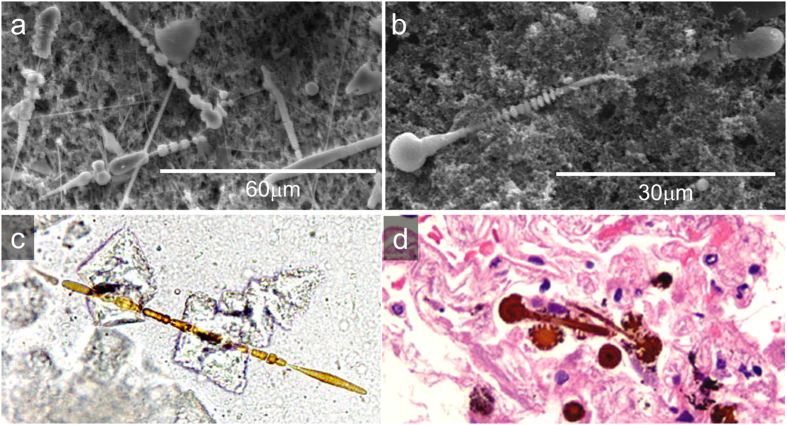
(**a**,**b**) Secondary electron SEM micrographs of d*AB*. (**c**) Optical microscopy image of an d*AB* extracted from lung tissue belonging to Case A (400x). (**d**) Optical microscopy image of h*AB* in a 3 μm-thick histological section stained with hematoxylin and eosin (H&E) belonging to Case A (400x).

At the optical microscope the *AB* appear as optically transparent fibres surrounded by a birifrangent golden-brown coat (Fig. 1c, and S1 in the Supporting Information (SI)). Secondary electron SEM images show that their overall diameter typically ranges between 1 and 4 μm, and their average length falls in the range between 20 and 120 μm. According to previous studies^7^,^10^, both optical microscopy (OM) and SEM images confirmed the typical features of the *AB*: their coating was often segmented along the fibre length into spaced spherical or ellipsoidal units, and often knobbed at the extremities^7^,^16^. SEM micrographs also show the inner crocidolite fibre in parts of the *AB* where the coating is interrupted (Figure S2 in the SI). The diameter of the fibres was measured to fall in the range between 0.3–0.4 μm, in agreement with that reported for crocidolite fibres^18^. Several uncoated fibres, were also detected (Fig. 1a and S1 in the SI). In the following, the abbreviation d*AB* will be used to refer to *AB* recovered from lung tissue after its chemical digestion and filtration on porous membranes, and h*AB* to the ones embedded in histological sections of the original lung tissue.

### Elemental quantification

Semi-quantitative EDS microanalysis was performed for preliminary characterization and to support XRF elemental quantification. The concentration of Fe measured by EDS was similar to that calculated by XRF (~27% wt. EDS vs. ~23% w/w XRF). Nevertheless, with the exception of Fe, all other elements detected by XRF were not detected by EDS. This can be explained by the fact that XRF analysis can reveal the presence of buried matter thanks to the higher probing depth of the X-rays compared to electrons^17^ (tens of μm vs. few μm, depending on the element considered and on the sample matrix). Spatially resolved elemental quantification with high lateral resolution was performed using XRF maps acquired on d*AB* at beamline ID18F (in air, at 14.4 keV), and maps acquired on d*AB* and h*AB* at beamline ID21 (in vacuum, at 7.3 keV). Representative XRF spectra acquired at the two beamlines are reported in Figure S3 in the SI. The elemental concentrations, reported in Table 1, were calculated by averaging the signal from pixels in selected areas of the *AB*, the lung tissue, or the background (see Methods and SI sections for more details). Since there were no significant differences between the results of Case A and Case B, elemental concentrations reported in Table 1 correspond to the weighted average of Cases A and Case B. The elemental composition of the h*AB* and d*AB* is compared with that of the lung tissue (TS), and with the ferritin (FR) and bovine liver (BL) references (Table 1). Elemental quantification of an empty membrane filtered with the same NaClO solution used to digest the lung tissue was performed to check for possible external contamination in the d*AB* (see spectrum Bkg2 in Figure S3 in the SI).

### Iron levels in the d*AB* and h*AB*

**Figure 2:**
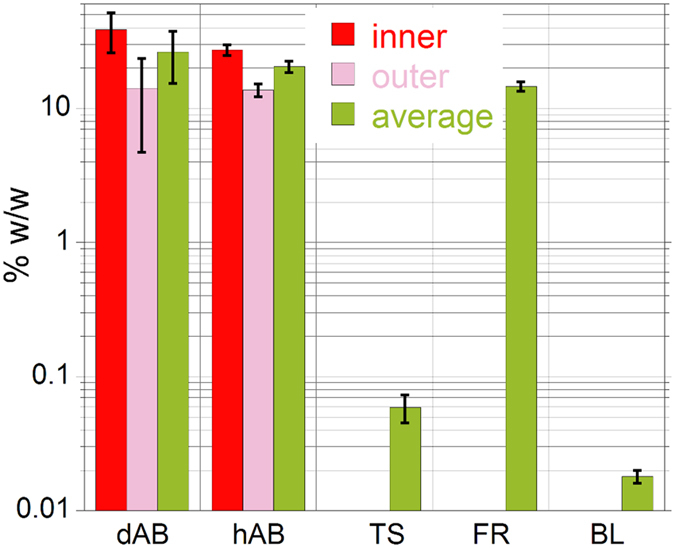
Iron concentrations in the inner and outer areas of the d*AB* and h*AB*, and their average values. Also shown for comparison are the Fe concentrations in lung tissue areas without *AB* (TS), and in the ferritin and bovine liver references (FR and BL, respectively). The error bars represent the average of the standard deviations reported in Table 1. The y-axis is in logarithmic scale.

**Figure 3:**
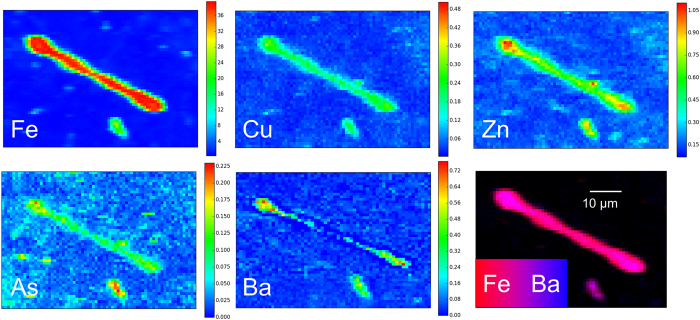
Elemental distribution maps of Fe, Cu, Zn, As, and Ba of a d*AB* (case A). The color bars indicate the elemental concentrations in % w/w. The distribution of the elements not associated with the *AB* (K and Ca) is reported in Figure S4 in the SI. The maps were acquired in air at 14.4 keV, with a pixel size is 2.5 × 2.0 μm^2^, and a dwell time of 1s.

**Figure 4:**
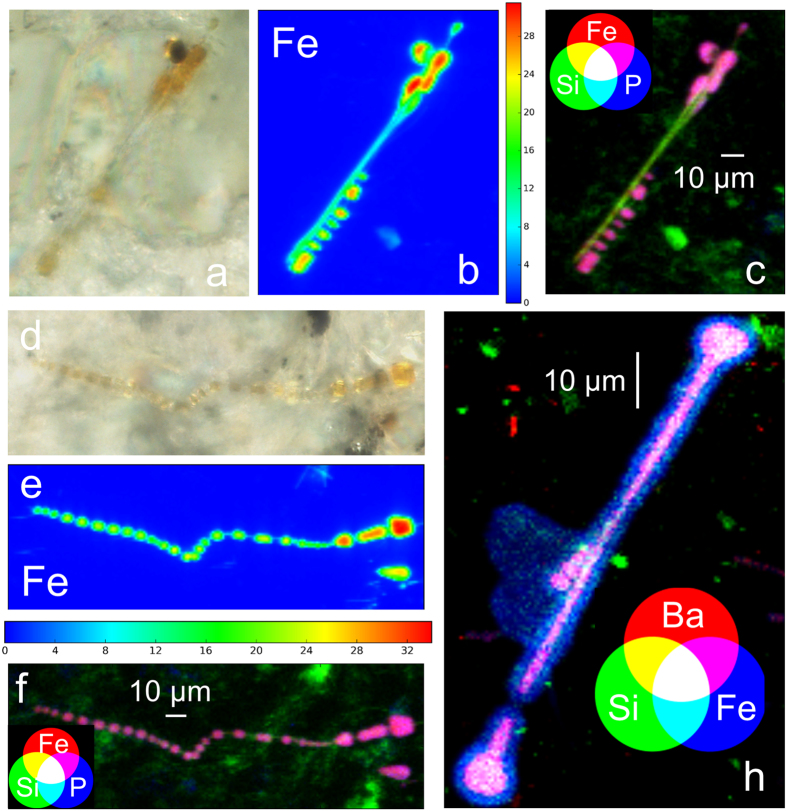
(**a**,**d**) Optical microscope images (500x) of h*AB* from case A and case B, respectively. (**b**,**e**) Elemental distribution maps of Fe in a h*AB* from case A and case B, respectively; the color bars indicate the concentration of Fe in % w/w. (**c**,**f**) RGB color combination showing the distribution of Si, Fe, and P of h*AB* from case A and case B, respectively. (**h**) Distribution and co-localization of Si, Fe, and Ba in a d*AB* from case A. The distributions of the other elements detected are reported in Figures S6 and S9 in the SI. The XRF maps shown in the Figure were acquired in vacuum at 7.3 keV, with a pixel size of 0.5 × 0.5 μm^2^, and a dwell time of 0.2s.

XRF elemental quantification indicated that Fe is by far the most concentrated element in the *AB*, exceeding the concentration of the other elements by up to three orders of magnitude (Table 1). Iron was found to be highly enriched in both d*AB* and h*AB* with respect to the Fe detected in the surrounding lung tissue (TS) and in the biological reference sample (BL) (~300x and ~1000x, respectively), in line with the qualitative results reported by Pascolo *et al*.^17^. As can be seen in Fig. 2 and Table 1, in all the investigated *AB* a significant difference in the concentration of Fe in the inner and outer areas was observed (Fig. 3). This is particularly evident in the higher resolution maps reported in Fig. 4. The Fe levels found in the outer part of the *AB* were similar to that measured for the ferritin standard (~14% w/w), and in agreement with the Fe concentration range reported for ferritin (10‒30 wt.% ref. 19). This finding supports the presence of Fe-storage proteins with high Fe-loading, as was already proposed^7^,^17^,^20^,^21^. On the other hand, the inner part of the *AB* contains from two to almost three times Fe (~27 to ~39% w/w) compared to the outer part. Similar trend for the Fe concentration was found for both the d*AB* and the h*AB* (Table 1). The average Fe concentration detected in the lung tissue (TS ~0.06% w/w) is of the same order of magnitude of that measured in the bovine liver reference (BL ~0.02% w/w), indicating that the tissue surrounding the *AB* is only slightly enriched in Fe with respect to the biological tissue reference. The concentration of Fe in the samples and references is summarized in the histogram shown in Fig. 2.

### Trace elements

Copper, Zn, As, and Ba were detected in the d*AB* in the 0.02‒0.3% w/w concentration range (Table 1). The presence of Cu and Zn is not surprising because they are well known essential physiological metals in human and animal organisms, and are present in several metallo-proteins and enzymes^22^. Arsenic, on the other hand, is widespread in the environment, in particular as a contaminant in groundwater^23^,^24^, and moderate As-contamination is common in the region from where the samples originate^25^,^26^. On the other hand, Ba may originate from barite (BaSO_4_), which is a common addictive in cement (the presence of Ba was previously reported on digested *AB* by means of ICP-MS analysis^22^). The presence of Cu, Zn, and As in the h*AB* cannot be confirmed due to the lower excitation energy used (7.3 keV), which was due to the technical features of beamline ID21. Quantification was not performed for elements lighter than Fe because large shifts were observed between nominal and experimental concentrations of the calibration standard below 5 keV. In maps acquired at 7.3 keV on h*AB*, quantification of K, P, and Ca was prevented also because of significant impurities of the same elements detected in the polyethylene-naphtalate (PEN) membrane used to support the histological sections (see spectrum Bkg2 in Figure S3 in the SI).

### Elemental Distribution

Representative XRF maps of d*AB* acquired at 14.4 and 7.3 keV are reported in Figs 3 and 4h, respectively. The distribution of the other elements detected is reported in Figures S4–S7 in the SI. The maps acquired at 14.4 keV (Fig. 3, and S4 and S5 in the SI) show that the spatial distribution of Fe, Cu, Zn, and As clearly mimics the morphology of the d*AB*, indicating either that those elements were involved in the biomineralisation process, such as Fe, or that they were adsorbed on the *AB* at some stage, which is probably the case of Cu, Zn, and As. In some d*AB*, lower elemental concentrations were observed inside the spherical lobes at both ends of the *AB*, suggesting a hollow structure (Figure S5 in the SI).

Elemental distribution maps of d*AB* and h*AB* acquired at higher resolution at ID21 beamline further confirm how Fe precisely reproduces the *AB* features, including the symmetric segmented sections perpendicular to the longitudinal axis and the rounded lobes at the extremities (Fig. 4). In vacuum experimental setup allowed for the detection of lighter elements, such as Si, P, and S. Maps acquired on the d*AB* revealed the presence of P, K, and Ca in small spots randomly distributed on the porous membranes (Figures S6 and S7 in the SI). In the h*AB*, those elements were instead observed co-localized with Fe (Fig. 4, S8, and S9 in the SI). Sulphur was only detected in the lung tissue surrounding the h*AB* (Figures S8 and S9 in the SI), where it is rather uniformly distributed. The distribution of Si and Fe in areas where the coating is missing or interrupted reveals the embedded asbestos fibres (Fig. 4), in agreement with the elemental composition of crocidolite asbestos, which is mainly made of Si, but also contain ~27 wt.% of Fe. Accordingly to SEM images, higher resolution maps confirmed that the fibres are about ten times thinner than their coating. It is interesting to note that single fibres seem to favour the deposition of the Fe-coating from the host organism with respect to bundled fibres (Fig. 4). Silicon is more concentrated in the inner part of the *AB*, matching the embedded asbestos fibre, but is also rather uniformly distributed at lower concentration on the entire *AB* area (Figures S6–S9 in the SI).

It is also interesting to note that the distribution of Ba differs from that of the other elements detected on the *AB*. Fluorescence maps acquired on d*AB* show that it is present in higher concentrations in the inner part of the *AB*, matching the area where the concentration of Fe is highest (Figs 4h, S6 and S7 in the SI). This is particularly evident in the map shown in Fig. 4h, in which the co-localization of Si, Fe, and Ba is highlighted by RGB colour combination.

### X-ray Absorption Spectroscopy

**Figure 5:**
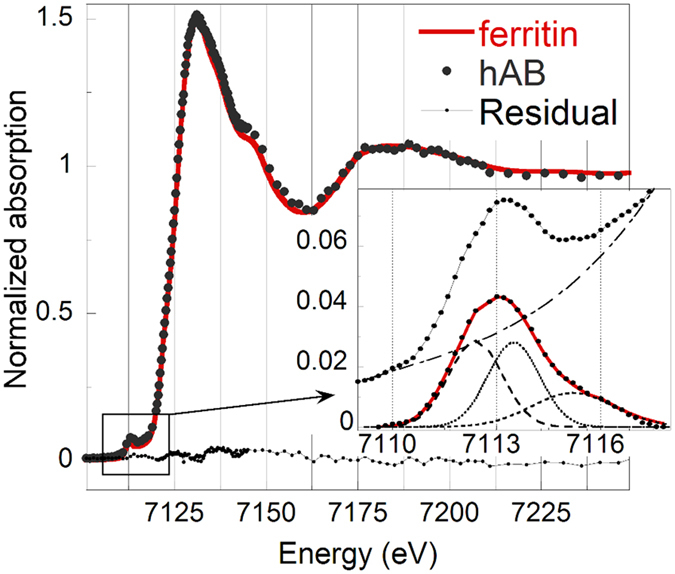
Background subtracted and normalized Fe K-edge XANES spectrum (average of 8 spectra) of a h*AB* compared with that of horse-spleen ferritin reference. The residual, A_ferritin_(E) − A_hAB_(E), is also shown to highlight the similarity of the two spectra. The h*AB* spectrum is the average of eight spectra acquired in different points of two different h*AB*. The inset shows the fit of the pre-edge peak performed using two pseudo-Voigt and one Gaussian functions, after subtraction of a baseline simulating the absorption edge.

X-ray absorption near edge structure (XANES) spectra of selected h*AB* and of several Fe reference compounds were acquired in an energy range between 7.0 and 7.3 keV, which includes the K absorption edge of Fe (7112 eV). Following the work of Wilke *et al*.^27^, after subtraction of an edge-shaped baseline, the pre-edge peaks of the XANES spectrum of a h*AB* were fitted with two pseudo-Voigt functions peaked at 7114.0 and 7115.1 eV, and a Gaussian function peaked at 7116.8 eV (Fig. 5, inset). The average energy position of the two lower energy peaks weighted for their areas is equal to 7114.6 eV, which, according to Wilke *et al*., indicates that Fe is in the 3+ oxidation state, compatibly with the presence of ferritin (or ferrihydrite). The unique features of the reference spectra (Figure S10 in the SI) indicate that it is possible to unambiguously identify the Fe speciation in the *AB* by fingerprint analysis of the XANES spectra (i.e. by comparison with the Fe reference spectra). The spectral features of the h*AB* spectrum match very well with those of the ferritin standard spectrum (Fig. 5). Linear combination fitting (LCF) using the spectra of Fe(III)-compounds was attempted to improve the match with the h*AB* spectrum. The match did not improved indicating that contributions from other Fe compounds, such as elemental iron or hematite, which were previously claimed to contribute up to ~20% to the XANES spectra of the *AB*^17^, can be excluded, at least in amounts larger than 5% (it is also worth noting that the presence of 1% of metallic iron, claimed in the same study, cannot be confirmed because the error associated with LCF procedure is in the range between 5 and 15%^28^,^29^,^30^). The XANES spectra of the h*AB* and of the ferritin standard are both very similar to the spectrum of the ferrihydrite standard (Figure S10 in the SI). This is not surprising, considering that the ferritin protein stores Fe at its interior in the form of ferrihydrite, and that, being XANES only sensitive to the local structure around the absorber atom (Fe, in the present case), it cannot distinguish between ferritin and ferrihydrite. On the other hand, the lack of a significant (>5%) contribution from the embedded crocidolite fibre, which was reported to contribute ~20% in a previous study^17^, can be explained by considering that the diameter of the fibre is about one-tenth that of its Fe-coating, and that the fluorescence signal scales accordingly.

## Discussion

Pulmonary alveolar macrophages are able to convert Fe into forms that can be retained indefinitely in the tissues. The iron proteins specifically dedicated to iron storage in human and animal organisms are ferritin and hemosiderin. Ferritin has several functions: it uptakes Fe^2+^, catalyses its oxidation to Fe^3+^, and limits its bioavailability to cell constituents^31^. Theoretically, a single ferritin molecule can contain up to 4500 iron atoms (i.e. 27 wt. %)^19^. The protein component of Fe-free ferritin, apoferritin, has a hollow spherical protein shell of outer diameter 12–13 nm and inner diameter of 7–8 nm. The cavity communicates with the surface by eight channels, which Fe can enter and leave. Iron enters in the form of Fe^2+^ and it is oxidized to Fe^3+^ as it is transferred into the core, where nucleation of ferrihydrite takes place. For this reason, pioneering studies exploiting x-ray and electron diffraction techniques^7^,^32^ identified the ferruginous coating as made of ferrihydrite. Hemosiderin, on the other hand, is formed by incomplete degradation of ferritin and conglomeration of iron and ferritin proteins, and differs from ferritin in having a higher iron-to-protein ratio. In addition, being less soluble in aqueous solutions, it represents a more stable and less available form of iron storage than ferritin. Under conditions of high Fe-excess, some of it may be stored in hemosiderin, whose formation may be favoured by the oxidative lung conditions^10^,^19^. Ferrihydrite, a poorly crystalline ferric oxide-hydroxide^33^,^34^, is abundant in a variety of aqueous geochemical environments and, because of its reactivity and large specific area, it has well known uptake ability towards As^35^,^36^ and Se^37^,^38^, Cr^39^, and U^40^,^41^, Ra and Ba^42^. It has also been reported that, at physiological pH and under oxidizing conditions, as in the lungs, ferrihydrite could preferentially uptake large ionic radius species, such Ra and Ba, metals, such as Pb, Cd, and Zn, and semi-metals such As and Se^22^. Elemental distribution maps acquired on d*AB* (Figs 3, and S4–S5 in the SI) confirmed that they are efficient scavengers for metals (Cu and Zn), semi-metals (As), and rare earths (Ba), in agreement with the affinity of ferrihydrite toward these species. Although Ba has been reported as a trace element in some asbestos samples^43^, and although its presence could be explained by the barite (BaSO_4_) used in production process of fibre-reinforced cement^44^, the lack of Ba in the h*AB* samples would suggest that it was introduced at some step of the digestion procedure (although Ba was not detected in the NaClO solution used to digest the lung tissue samples as can be seen from the corresponding XRF spectrum, Bkg1, in Figure S3 in the SI). Similarly, since the presence of Cu, Zn, and As in the h*AB* could not be confirmed, due to the lower excitation energy used (7.3 keV), it cannot be excluded that their presence could also be due to external contamination occurred during the digestion step, and to their subsequent enrichment on the *AB* during the filtration through the porous membranes. Further measurements on h*AB* at higher energies would help settling this point.

In d*AB* maps, K, P, and Ca were found in small agglomerates randomly distributed on the porous membranes, where residual lung tissue accumulated (Figures S4–S7 in the SI), while in h*AB* maps those elements were found associated with the *AB*, co-localized with Fe. This would suggest that they were present as soluble species on the *AB* and then removed during the digestion with NaClO. While P and Ca have already been observed in the *AB* in previous works, claiming their active role in the biomineralization process^16^,^17^, the presence of K associated with the fibres’ coating has never been directly observed before.

High resolution fluorescence maps (Fig. 4) show that Si is more concentrated in the inner part of the *AB*, revealing the inner asbestos fibre, but it is also spread at lower concentration over the entire *AB* area, suggesting the incipient dissolution of the fibre itself. The possible Degradation of asbestos fibres after long residence time in the lungs is a matter of a long standing debate. This subject is of interest because it is widely accepted that among the reasons for the high carcinogenicity of asbestos fibres there are, on one hand, their high biopersistence in biological tissues, and, on the other hand, the possibility that the elemental constituents of the fibres are released to the surrounding tissues upon degradation of the fibres itself^45^. The elemental distribution observed in this work is in agreement with recent observation by high resolution TEM of the formation of silica-rich amorphous coatings around asbestos tremolite-actinolite fibres^46^. The authors suggested that these coatings are associated with the dissolution of the amphiboles, and observed that silicon is the last to dissolve as the coatings progressively accumulates. They therefore conclude that solid state diffusion of Ca, Fe, and Mg ions out of the crystal lattice of the fibres results in a silica residue that eventually replaces the fibre by alteration.

**Figure 6:**
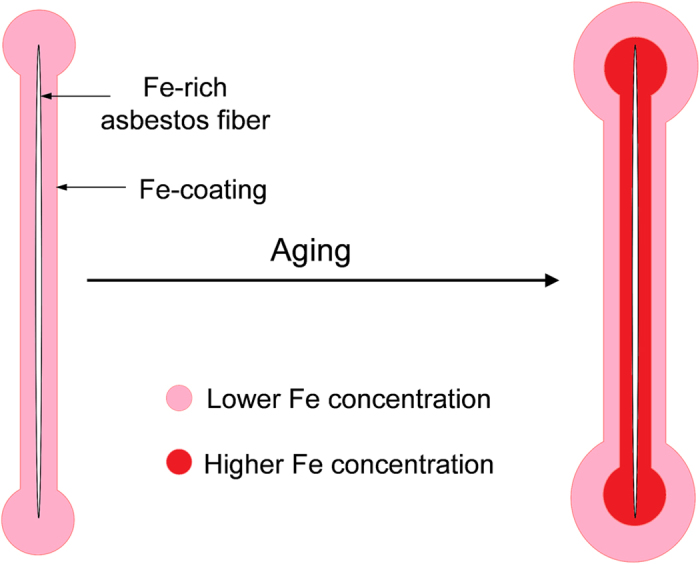
Speculative model proposed to explain the observed distribution of Fe in the asbestos bodies.

Altogether, the observed distribution of Si and Fe suggests a possible model for the evolution of the *AB* during prolonged residence in the lung tissue. As demonstrated by *in vitro* studies^47^,^48^, alveolar macrophages start to deposit endogenous Fe on the asbestos fibres soon after their injection in the host tissue, and XANES results confirmed that Fe is deposited in the form of ferrihydrite, the mineral core of ferritin and hemosiderin. The concentration of Fe in the inner part of the *AB* is higher with respect to the more recently deposited external layer. This may be attributed to the gradual conversion of the primarily deposited and Fe-overloaded ferritin in the inner part of the *AB*, into hemosiderin with higher Fe content, and to subsequent precipitation of its ferrihydrite core (the presence of hemosiderin in the *AB* has been proposed already in 1965^49^, but its actual presence could not be confirmed due to difficulty of distinguishing between ferritin and hemosiderin). An alternative explanation for the observed distribution of Fe may arise from the release of exogenous Fe from the asbestos fibre itself, which would undergo to gradual dissolution, as the observed distribution of Si would suggest. The Fe distribution may also be determined by the combination of the above-mentioned mechanisms, one being a consequence of the other: Fe-excess originating from the dissolution of the fibre may induce the conversion of ferritin into hemosiderin. In the framework of this model, the observed peculiar distribution of Ba could be determined by possible differences in the uptake abilities of ferritin and hemosiderin. The model describing the observed data is depicted in the sketch shown in Fig. 6. It is worth noting that the model remains speculative, as it is not possible to confirm it in laboratory time-scale experiments.

The comparison of the results obtained on the d*AB* and on the h*AB*, allowed unambiguously distinguishing the composition of the *AB* from that of the lung tissue, and understanding to what extent the digestion procedure altered their chemical composition. In particular, such comparison suggested that species previously reported to be associated with the *AB*^22^ (such as Ba), may have been introduced by external contamination during the digestion of the biological tissue, and subsequently migrated in the *AB*, which were proven to be excellent scavengers for elements having high affinity with ferrihydrite. Phosphorus, K, and Ca, on the other hand, were found to be associated with the AB coating, but were dissolved and removed by chemical digestion with NaClO. Finally, the comparison between the XANES spectra of the *AB* and of ferritin, indicated that hematite and metallic iron, whose presence in the *AB* was claimed in a previous study^17^, are absent or present in amounts well below 5%, and thus that the *AB* are mainly composed by ferritin and/or hemosiderin.

## Methods

### Samples

Human lung samples were collected after forensic autopsy from two former workers of an asbestos plant in North-West Italy. Both cases were affected by pulmonary asbestosis; case A had also pleural mesothelioma, while case B had also lung cancer. The grade of asbestosis (Table 2), was established according to Craighead *et al*.^50^. Accordingly to the type of asbestos used in the plant, EDS microanalysis of uncoated parts of the fibres (Figure S2 in the SI) allowed identifying them as crocidolite asbestos, which has ideal chemical formula NaFe_2_(Fe, Mg)_3_Si_8_O_22_(OH)_2_ and contain ~27% Fe^18^. More detailed information on the nature of asbestos fibres from samples with the same origin can be found in a previous work^51^. Lung samples were preserved in formalin (10%) until non-neoplastic portions (0.25 g) of lung tissue were digested in 30 mL sodium hypochlorite solution (NaClO, reagent grade, chlorine content 10–15%, Merck) to dissolve the organic matrix. The suspension of inorganic material was filtered through mixed cellulose esters porous membranes (pore size 0.45 μm, Millipore) to recover the *AB*. The membranes were then thoroughly washed with warm (40 °C) deionized water to dissolve NaCl crystals formed during digestion, and finally air-dried. SEM and light microscope images of these samples (d*AB*) are shown in Fig. 1, and S1 in the SI. Further portions of non-neoplastic lung tissues were collected to prepare 3 and 10 μm-thick histological sections with a microtome. To avoid interference during XRF experiments and increase the probability to locate the *AB*, 10 μm-thick non-stained histological sections were fixed on polyethylene-naphtalate (PEN) membranes (from MMI). Due to the extreme difficulty experienced in locating the *AB* embedded in the lung tissue, a laser micro-dissector (Nikon) coupled with an optical microscope was used to cut 100 μm-diameter areas of the histological sections centred on the *AB*. Light microscope images of the *AB* in the cut sections are shown in Fig. 4a and d.

Three μm-thick sections were embedded in paraffin, fixed on standard microscope slides, and then stained with haematoxylin and eosin, according to the standard protocol^52^, for histological examination. Lung samples were examined to estimate the number of *AB* by optical microscope (Leica DMLB) and SEM (Cambridge Stereoscan S360). According to the international standard^53^, the concentration of *AB* was expressed as their number per gram of dry weight (g_dw_). This quantity was derived following the procedure described in Belluso *et al*.^54^: (i) the whole membrane was observed by optical microscopy at 400x magnifications, and (ii) a portion of the membrane representing 0.7% of its total area (about 2 mm^2^) was observed by SEM at 2000x magnifications. The equivalent dry weight was calculated by dehydrating 2.5 g of wet tissue of each specimen at 60 °C for 3 days. In both specimens the burden of *AB* largely exceeded the amount established by the European Respiratory Society guidelines (10^3^/g_dw_) to indicate a high level of occupational exposure to asbestos^53^ (Table 2).

### Synchrotron experiments

Several *AB* from each human subject were analysed during each of the three synchrotron experiments performed at two different beamlines at the ESRF synchrotron facility (Table S3). Different experimental conditions (namely, in-vacuum or in-air acquisitions, 7.3 or 14.4 keV incident photon energy, and different beam spot-size, Table S3 in the SI) were dictated by the technical features of the beamlines, and allowed to obtain complementary information on the samples (i.e. elements lighter or heavier than Fe). d*AB* samples supported on cellulose esters membranes and h*AB* samples supported on PEN membranes were mounted on dedicated sample holders, covered with 4 μm-thick Ultralene^®^ polymer film, and measured using solid state fluorescence detectors in 45°/45° geometry. Detailed information on the beamlines technical features and on the experimental setups is reported in the SI.

### Elemental quantification

Semi-quantitative EDS measurements were performed with a Si(Li) detector (Link-Oxford Pentafet ATW2) coupled with the SEM, in vacuum (~10^−5 ^mbar) and at electron accelerating voltage of 15 kV. A Co standard was used to check the stability of the incident beam. The electron beam excitation area and penetration depth were estimated to be of ~1 μm^2^ and <1 μm, respectively. Absolute elemental quantification by XRF was performed on the fluorescence maps acquired on d*AB* at the ID18F beamline and on h*AB* at the ID21 beamline at the ESRF, at incident photon energies of 14.4 and 7.3 keV, respectively. Fluorescence signal of each detected element was fitted and deconvoluted using the PyMCA software package^55^, which also allows defining the absorbing matrix and taking into account absorbers between the sample and the detector (air, windows, filters, and others). Quantification was performed by manually selecting the pixels of the areas of interest (inner or outer *AB* area, whole AB area, and whole map area for the references), and averaging the fluorescence signals of each pixel. Great attention was paid to the choice of the calibration standards and to the definition of the absorbing matrix (see SI). Certified reference standards included SRM1832-1833 and bovine liver (SRM1577b) from NIST, and a reference standard composed by seven ultra-thin layers of different metals (Pd, Ca, La, Fe, Cu, Pb, and Mo) sputtered on a 0.2 μm-thick silicon nitride membrane from AXO-Dresden, were used. A ferritin reference (F7879, Sigma-Aldrich) was prepared by dropping and drying a small amount of the ferritin solution on a Kapton film. The working energies and experimental conditions allowed to detect elements from Si to Br (K-edges), and from Ru to Pb (L-edges), but elemental quantification was considered reliable only for elements heavier than Ca. Further details on the quantification procedure (definition of the absorbing matrix, data processing, and analysis) can be found in the SI.

### Elemental distribution

High resolution fluorescence maps (pixel size 0.5 × 0.5 μm^2^) were acquired on the d*AB* and h*AB* in vacuum (10^−5 ^mbar), at incident photon energy of 7.3 keV at the ID21 beamline at the ESRF. The excitation energy and the in vacuum setup allowed for the detection of elements from Na to Fe (K-edges) and from Rb to Nd (L-edges). Fluorescence maps acquired on d*AB* at ID18F have lower resolution (2.5 × 2.0 μm^2^), but allowed for the detection of elements heavier than Fe (K-edges from Co to Br).

### X-ray Absorption Spectroscopy

XAS is a chemical selective tool informative of the oxidation state and of the chemical and crystallographic neighbourhood of a selected element^56^. Several XANES spectra were acquired at the Fe K-edge at the ID21 beamline on different points of selected h*AB* using beam spot sizes of 0.5 × 0.5 μm^2^. Reference XANES spectra of commercial horse spleen ferritin (Sigma-Aldrich) and of other relevant Fe commercial compounds (magnetite, hematite, hemin, haematin, and Fe(II)-L-ascorbate by Sigma-Aldrich, and Fe metallic foil by MaTeck) were measured along with the samples (Figure S10 in the SI). The 2-lines ferrihydrite and crocidolite UICC standard reference spectra were kindly granted by Dr. A. Voegelin and Prof. A. Gualtieri, respectively. XANES spectra were energy calibrated with respect to the spectrum of an iron foil, and background subtracted and normalized using the IFEFFIT software package^57^. Further details on the Linear Combination Fitting (LCF) and pre-edge peaks analyses of the XANES spectra are reported in the SI.

All methods were carried out in accordance with relevant guidelines and regulations, and all experimental protocols were approved by the Bioethical committees of the Martini hospital (Turin, Italy) and of the University of Torino (Turin, Italy). Informed consent was obtained from the subjects at the moment of their hospitalization for generic research purposes.

## Additional Information

**How to cite this article**: Bardelli, F. *et al*. New insights on the biomineralisation process developing in human lungs around inhaled asbestos fibres. *Sci. Rep.* **7**, 44862; doi: 10.1038/srep44862 (2017).

**Publisher's note:** Springer Nature remains neutral with regard to jurisdictional claims in published maps and institutional affiliations.

## Supplementary Material

Supplementary Information

## Figures and Tables

**Table 1:** XRF elemental quantification (% w/w) of d*AB* and h*AB*, lung tissue (*TS*), and of the ferritin (*FR*) and bovine liver (*BL*) standards.

	^a^d*AB*		^b^h*AB*			^c^*FR*	^d^*BL*
	^e^Inner	^f^Outer	^e^Inner	^f^Outer	^g^*TS*		
Fe	38.7 ± 12.8	14.1 ± 9.4	27.3 ± 2.5	13.7 ± 1.5	0.059 ± 0.014	14.6 ± 1.2	0.018 ± 0.002
Cu	0.06 ± 0.03	0.04 ± 0.01				nd	0.021 ± 0.002
Zn	0.31 ± 0.17	0.22 ± 0.06				0.017 ± 0.001	0.016 ± 0.001
As	0.05 ± 0.02	0.02 ± 0.01				0.013 ± 0.003	nd
Ba	0.22 ± 0.08	0.072 ± 0.012	nd	nd	nd	nd	nd

Data belonging to Cases A and B are reported averaged (weighted average). Elemental quantification of d*AB* and standards was obtained from measurements performed at incident photon energy of 14.4 keV at ID18F, while that of h*AB* was performed on data acquired at 7.3 keV at ID21, therefore no information on Cu, Zn, and As is available for the latter. The errors represent the standard deviations on *N* XRF measurements (*N* = 9 for the d*AB* (5 for Case A, 4 for Case B), and *N* = 8 for the h*AB* and the TS (4 for Case A, and 4 for Case B). See SI for details about the estimation of the absolute uncertainties. “nd” stands for not detected or below the 0.001% w/w detection limit.

^a^*AB* deposited on cellulose esters porous membranes upon filtering the chemically digested lung tissue; ^b^*AB* in paraffin-embedded histological lung sections; ^c^Horse-spleen ferritin reference (F7879 from Sigma-Aldrich); ^d^Bovine liver certified standard (SRM1577b from NIST); ^e^average of the XRF signal of the pixels corresponding to the inner *AB* area; ^f^average of the XRF signal of the pixels corresponding to the outer *AB* area; ^g^measurements performed on areas of the histological sections without *AB*.

**Table 2:** Samples’ description.

Case	Age	Sex	Occupation	Exposure period	Asbestos type	Disease	*AB* count (/g_dw_)
A	81	M	Fibre cement plant	27 years	Crocidolite	^a^AS (grade 3), ^b^PP, ^c^MM	~3.6·10^5^
B	80	F	Fibre cement plant	unknown	Crocidolite	AS (grade 4), PP, ^d^LC	~1.2·10^6^

^a^AS: Asbestosis; ^b^PP: pleural plaques; ^c^MM: pleural mesothelioma; ^d^LC: lung cancer.

## References

[b1] Mossman B. T. *In vitro* studies on the biologic effects of fibers: correlation with *in vivo* bioassays. Environ. Health Perspect. 88, 319–22 (1990). 2272329 10.1289/ehp.9088319 PMC1568030

[b2] Warnock M. L. & Churg A. M. Association of asbestos and bronchogenic carcinoma in a population with low asbestos exposure. Cancer 35, 1236–42 (1975). 1116110 10.1002/1097-0142(197504)35:4<1236::aid-cncr2820350431>3.0.co;2-5

[b3] Price B. & Ware A. Mesothelioma trends in the United States: an update based on Surveillance, Epidemiology, and End Results Program data for 1973 through 2003. Am. J. Epidemiol. 159, 107–12 (2004). 14718210 10.1093/aje/kwh025

[b4] Robinson B. W. S. & Lake R. A. Advances in Malignant Mesothelioma. New Engl. J. Med. 353, 1591–1603 (2005). 16221782 10.1056/NEJMra050152

[b5] Asbestos Disaster - Lessons from Japan’s Experience | Kenichi Miyamoto | Springer. Available at: http://www.springer.com/us/book/9784431539148 (Accessed: 19th May 2016).

[b6] Churg A. M. & Warnock M. L. Asbestos and other ferruginous bodies: their formation and clinical significance. Am. J. Pathol. 102, 447–56 (1981). 6101235 PMC1903711

[b7] Pooley F. D. Asbestos bodies, their formation, composition and character. Environ. Res. 5, 363–79 (1972). 4568594 10.1016/0013-9351(72)90039-4

[b8] Mace M. L., McLemore T. L., Roggli V., Brinkley B. R. & Greenberg S. D. Scanning electron microscopic examination of human asbestos bodies. Cancer Lett. 9, 95–104 (1980). 7379046 10.1016/0304-3835(80)90112-3

[b9] McLemore T. L. . Asbestos body phagocytosis by human free alveolar macrophages. Cancer Lett. 9, 85–93 (1980). 7379045 10.1016/0304-3835(80)90111-1

[b10] Ghio A. J., Churg A. & Roggli V. L. Ferruginous bodies: implications in the mechanism of fiber and particle toxicity. Toxicol. Pathol. 32, 643–9 (2004). 15513907 10.1080/01926230490885733

[b11] Governa M. & Amati M. Role of iron in Asbestos-Body-induced oxidant radical generation. J. Toxicol. Environ. Heal. Part A 58, 279–287 (1999). 10.1080/009841099157241 10598953

[b12] Fubini B. & Mollo L. Role of iron in the reactivity of mineral fibers. Toxicol. Lett. 82–83, 951–60 (1995). 10.1016/0378-4274(95)03531-1 8597167

[b13] Lund L. G., Williams M. G., Dodson R. F. & Aust, A. E. Iron associated with asbestos bodies is responsible for the formation of single strand breaks in phi X174 RFI DNA. Occup. Environ. Med. 51, 200–204 (1994). 8130850 10.1136/oem.51.3.200 PMC1127940

[b14] Gianoncelli A. . Life science applications and research potential of the TwinMic spectromicroscopy station at ELETTRA. J. Phys. Conf. Ser. 463, 12004 (2013).

[b15] Ide-Ektessabi A. Applications of Synchrotron Radiation - Micro Beams in Cell Micro Biology (Springer Berlin Heidelberg, 2007).

[b16] Pascolo L. . Synchrotron soft X-ray imaging and fluorescence microscopy reveal novel features of asbestos body morphology and composition in human lung tissues. Part. Fibre Toxicol. 8, 7 (2011). 21299853 10.1186/1743-8977-8-7 PMC3041679

[b17] Pascolo L. . The interaction of asbestos and iron in lung tissue revealed by synchrotron-based scanning X-ray microscopy. Sci. Rep. 3, 1123 (2013). 23350030 10.1038/srep01123 PMC3553542

[b18] Hawthorne F. C. & Oberti R. Classification of the Amphiboles. Rev. Mineral. Geochemistry 67, 55–88 (2007).

[b19] Harrison P. & Arosio P. The ferritins: molecular properties, iron storage function and cellular regulation. Biochim. Biophys. Acta 1275, 161–203 (1996). 8695634 10.1016/0005-2728(96)00022-9

[b20] Borelli V. . A procedure for the isolation of asbestos bodies from lung tissue by exploiting their magnetic properties: a new approach to asbestos body study. J. Toxicol. Environ. Health. A 70, 1232–40 (2007). 17573637 10.1080/15287390701380906

[b21] Ghio A. J., Stonehuerner J., Richards J. & Devlin R. B. Iron homeostasis in the lung following asbestos exposure. Antioxid. Redox Signal. 10, 371–7 (2008). 17999626 10.1089/ars.2007.1909

[b22] Nakamura E., Makishima A., Hagino K. & Okabe K. Accumulation of radium in ferruginous protein bodies formed in lung tissue: association of resulting radiation hotspots with malignant mesothelioma and other malignancies. Proc. Japan Acad. Ser. B 85, 229–239 (2009). 19644223 10.2183/pjab.85.229 PMC3561846

[b23] Bardelli F. . Arsenic uptake by natural calcite: An XAS study. Geochim. Cosmochim. Acta 75, 3011–3023 (2011).

[b24] Winkel L., Casentini B. & Bardelli F. Speciation of arsenic in Greek travertines: Co-precipitation of arsenate with calcite. Geochim. Cosmochim. Acta 106, 99 (2013).

[b25] Marengo E. . Statistical analysis of ground water distribution in Alessandria Province (Piedmont—Italy). Microchem. J. 88, 167–177 (2008).

[b26] Caviglia C., Destefanis E., Masciocco L. & Re D. Environmental problems related to the presence of arsenic in the Anza Valley (Piedmont, north-western Italy). Eng. Geol. Soc. Territ. 3, 421–424 (2014).

[b27] Wilke M., Farges F., Petit P. E., Brown G. E. & Martin F. Oxidation state and coordination of Fe in minerals: An FeK- XANES spectroscopic study. Am. Mineral. 86, 714–730 (2001).

[b28] Bardelli F., Cattaruzza E., Gonella F., Rampazzo G. & Valotto G. Characterization of road dust collected in Traforo del San Bernardo highway tunnel: Fe and Mn speciation. Atmos. Environ. 45, 6459–6468 (2011).

[b29] Isaure M.-P. . Quantitative Zn speciation in a contaminated dredged sediment by μ-PIXE, μ-SXRF, EXAFS spectroscopy and principal component analysis. Geochim. Cosmochim. Acta 66, 1549–1567 (2002).

[b30] Varrica D., Bardelli F., Dongarrà G. & Tamburo E. Speciation of Sb in airborne particulate matter, vehicle brake linings, and brake pad wear residues. Atmos. Environ. 64, 18–24 (2013).

[b31] Chasteen N. D. & Harrison P. M. Mineralization in ferritin: an efficient means of iron storage. J. Struct. Biol. 126, 182–94 (1999). 10441528 10.1006/jsbi.1999.4118

[b32] Shen Z. . Using *in vitro* iron deposition on asbestos to model asbestos bodies formed in human lung. Chem. Res. Toxicol. 13, 913–21 (2000). 10995265 10.1021/tx000025b

[b33] Michel F. M. . Reactivity of ferritin and the structure of ferritin-derived ferrihydrite. Biochim. Biophys. Acta 1800, 871–85 (2010). 20510340 10.1016/j.bbagen.2010.05.007

[b34] Drits V. A., Sakharov B. A., Salyn A. L. & Manceau A. Structural model for ferrihydrite. Clay Miner. 28, 185–207 (1993).

[b35] Mähler J. & Persson I. Rapid adsorption of arsenic from aqueous solution by ferrihydrite-coated sand and granular ferric hydroxide. Appl. Geochemistry 37, 179–189 (2013).

[b36] Raven K. P., Jain A., Raven R. H. L., Jain K. P. A. & Loeppert R. H. Arsenite and arsenate adsorption on ferrihydrite: kinetics, equilibrium, and adsorption envelopes. Environ. Sci. Technol. 32, 344–349 (1998).

[b37] Voegelin A., Kaegi R., Frommer J., Vantelon D. & Hug S. J. Effect of phosphate, silicate, and Ca on Fe(III)-precipitates formed in aerated Fe(II)- and As(III)-containing water studied by X-ray absorption spectroscopy. Geochim. Cosmochim. Acta 74, 164–186 (2010).

[b38] Hayes K. F. . *In Situ* X-ray Absorption Study of Surface Complexes: Selenium Oxyanions on agr-FeOOH. Science 238, 783–6 (1987). 17814706 10.1126/science.238.4828.783

[b39] Loyaux-Lawniczak S., Refait P., Ehrhardt J.-J., Lecomte P. & Génin J.-M. R. Trapping of Cr by Formation of Ferrihydrite during the Reduction of Chromate Ions by Fe(II)−Fe(III) Hydroxysalt Green Rusts. Environ. Sci. Technol. 34, 438–443 (2000).

[b40] Hsi C. D. & Langmuir D. Adsorption of uranyl onto ferric oxyhydroxides: Application of the surface complexation site-binding model. Geochim. Cosmochim. Acta 49, 1931–1941 (1985).

[b41] Stubbs J. E., Elbert D. C., Veblen D. R. & Zhu C. Electron Microbeam Investigation of Uranium-Contaminated Soils from Oak Ridge, TN, USA Environ. Sci. Technol. 40, 2108–2113 (2006). 16646440 10.1021/es0518676

[b42] Sajih M. . Adsorption of radium and barium on goethite and ferrihydrite: A kinetic and surface complexation modelling study. Geochim. Cosmochim. Acta 146, 150–163 (2014).

[b43] Upreti R. K., Dogra R. K. S., Shanker R., Krishna Murti C. R., Dwivedi K. K. & Rao G. N. Trace elemental analysis of asbestos with an x-ray fluorescence. Sci. Total Environ. 40, 259–276 (1984).

[b44] Ling T.-C. & Poon C.-S. High temperatures properties of barite concrete with cathode ray tube funnel glass. Fire Mater. 38, 279–289 (2014).

[b45] Pollastri S. . The chemical environment of iron in mineral fibres. A combined X-ray absorption and Mössbauer spectroscopic study. J. Hazard. Mater. 298, 282–293 (2015). 26073382 10.1016/j.jhazmat.2015.05.010

[b46] Germine M. & Puffer J. H. Analytical Transmission Electron Microscopy of Amphibole Fibers from the Lungs of Quebec Miners. Arch. Environ. Occup. Health, doi: 10.1080/19338244.2014.918928 (2014). 25386835

[b47] Koerten H. K., Hazekamp J., Kroon M. & Daems W. T. Asbestos Body Formation and Iron Accumulation in Mouse Peritoneal Granulomas After the Introduction of Crocidolite Asbestos Fibers. 136 (1990). PMC1877471 2153345

[b48] Koerten H. K., de Bruijn J. D. & Daems W. T. The formation of asbestos bodies by mouse peritoneal macrophages. An *in vitro* study. Am. J. Pathol. 137, 121–34 (1990). 2372038 PMC1877687

[b49] Richter G. W. & Bessis M. C. Commentary on hemosiderin. Blood 25, 370–374 (1965). 14263211

[b50] Craighead J. E. . The pathology of asbestos-associated diseases of the lungs and pleural cavities: diagnostic criteria and proposed grading schema. Report of the Pneumoconiosis Committee of the College of American Pathologists and the National Institute for Occupational Sa. Arch. Pathol. Lab. Med. 106, 544–96 (1982). 6897166

[b51] Magnani C. . Asbestos lung burden and asbestosis after occupational and environmental exposure in an asbestos cement manufacturing area: a necropsy study. Occup. Environ. Med. 55, 840–846 (1998). 9924446 10.1136/oem.55.12.840 PMC1757537

[b52] Luna L. Manual of histologic staining methods of the Armed Forces Institute of Pathology (Blakiston Division McGraw-Hill, 1968).

[b53] De Vuyst P. . Guidelines for mineral fibre analyses in biological samples: report of the ERS Working Group. European Respiratory Society. Eur. Respir. J. Off. J. Eur. Soc. Clin. Respir. Physiol. 11, 1416–26 (1998). 10.1183/09031936.98.11061416 9657589

[b54] Belluso E. . Assessment of Inorganic Fibre Burden in Biological Samples by Scanning Electron Microscopy – Energy Dispersive Spectroscopy. Microchim. Acta 155, 95–100 (2006).

[b55] Solé V. A., Papillon E., Cotte M., Walter P. & Susini J. A multiplatform code for the analysis of energy-dispersive X-ray fluorescence spectra. Spectrochim. Acta Part B At. Spectrosc. 62, 63–68 (2007).

[b56] Lee P. A., Citrin P. H., Eisenberger P. & Extendend K. B. x-ray absorption fine structure-its strenghts and limitations as a structural tool. Rev. Mod. Phys. 53, 769–806 (1981).

[b57] Ravel B. & Newville M. ATHENA, ARTEMIS, HEPHAESTUS: data analysis for X-ray absorption spectroscopy using IFEFFIT. J. Synchrotron Radiat. 12, 537–41 (2005). 15968136 10.1107/S0909049505012719

