# Supplementary material for: New insights on the biomineralisation process developing in human lungs around inhaled asbestos fibres
Source: Sci Rep. 2017 Mar 23;7:44862. doi: 10.1038/srep44862 (PMC5362951; doi:10.1038/srep44862)
Supplement: Supplementary Information [file srep44862-s1.pdf]

New insights on the biomineralisation process developing in human lungs around inhaled asbestos fibres

Fabrizio Bardelli, Giulia Veronesi, Silvana Capella, Donata Bellis, Laurent Charlet, Alessia Cedola, and Elena Belluso

Supporting Information

Index

Supporting Tables and Figures

SEM and OM images of the samples 1

Representative X-ray fluorescence spectra 4

Additional elemental distribution maps 5

X-ray Absorption Spectroscopy (XAS) spectra of samples and references 11

Supporting Information to the Methods section

Additional details on the synchrotron radiation experiments 12

Additional details on the elemental quantification 12

Additional details on XAS acquisitions and data analysis 13

References 14

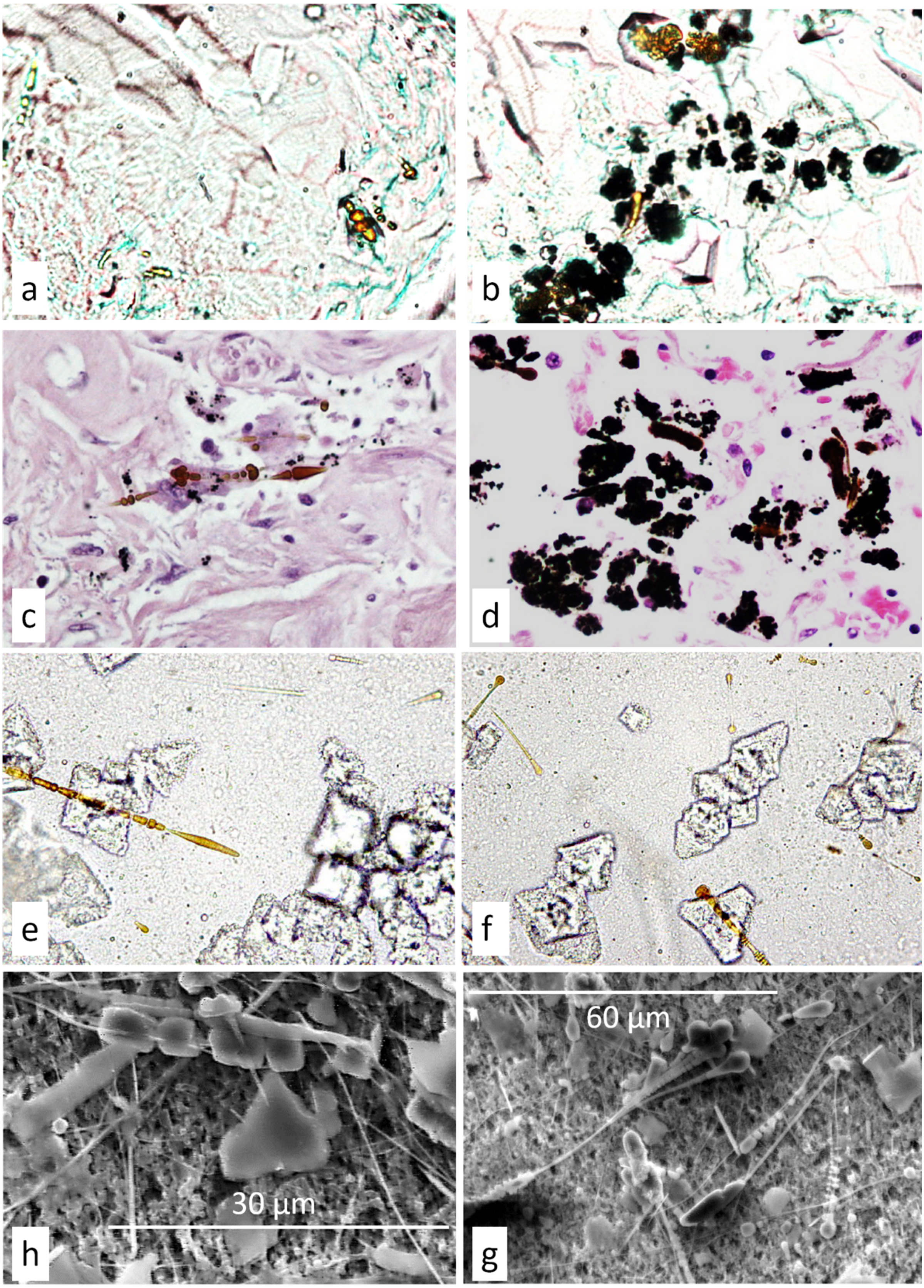

Figure S1. Panels a) and b): OM images (400x) of h*AB* embedded in non-stained 10 µm-thick histological sections. Panels c) and d): OM images (400x) of h*AB* embedded in H&E-stained 3 µm-thick histological sections. Panels e) and f): OM images (400x) of d*AB* deposited on porous membranes upon digestion and filtration of the biological tissue. Panels h) and g) SEM images of d*AB* deposited on porous membranes upon digestion and filtration of the biological tissue. Panels a), c), e), h) correspond to samples belonging to Case A, while the ones reported in panels b), d), f), g) belong to Case B.

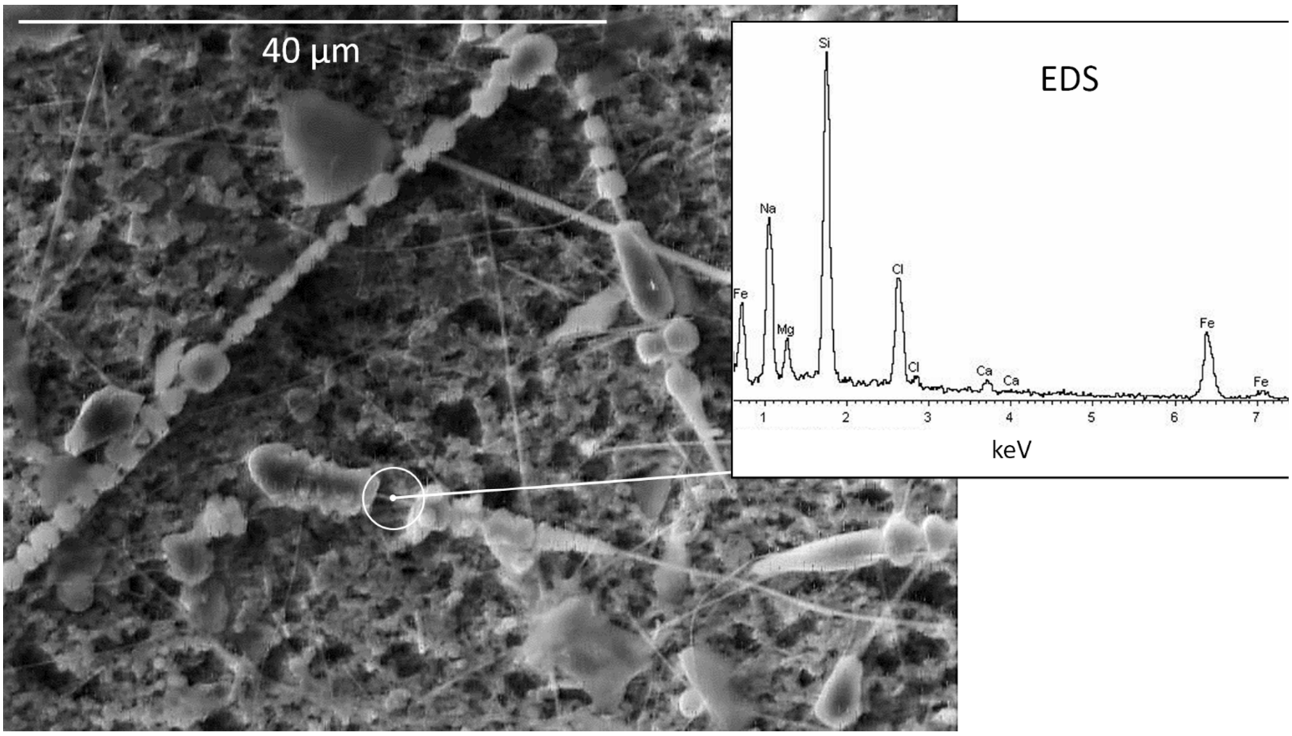

Figure S2. EDS spectrum of a point acquisitions performed on the uncoated part of a d*AB* indicated in the SEM image. The elemental composition is compatible with the ideal chemical formula of crocidolite asbestos[1], except for an excess of Na and Cl, which partly originates from the NaCl crystals formed after chemical digestion with NaClO and not fully removed by washing at 40°C with deionized water. The nominal electron beam spot size was ~1 μm^2.

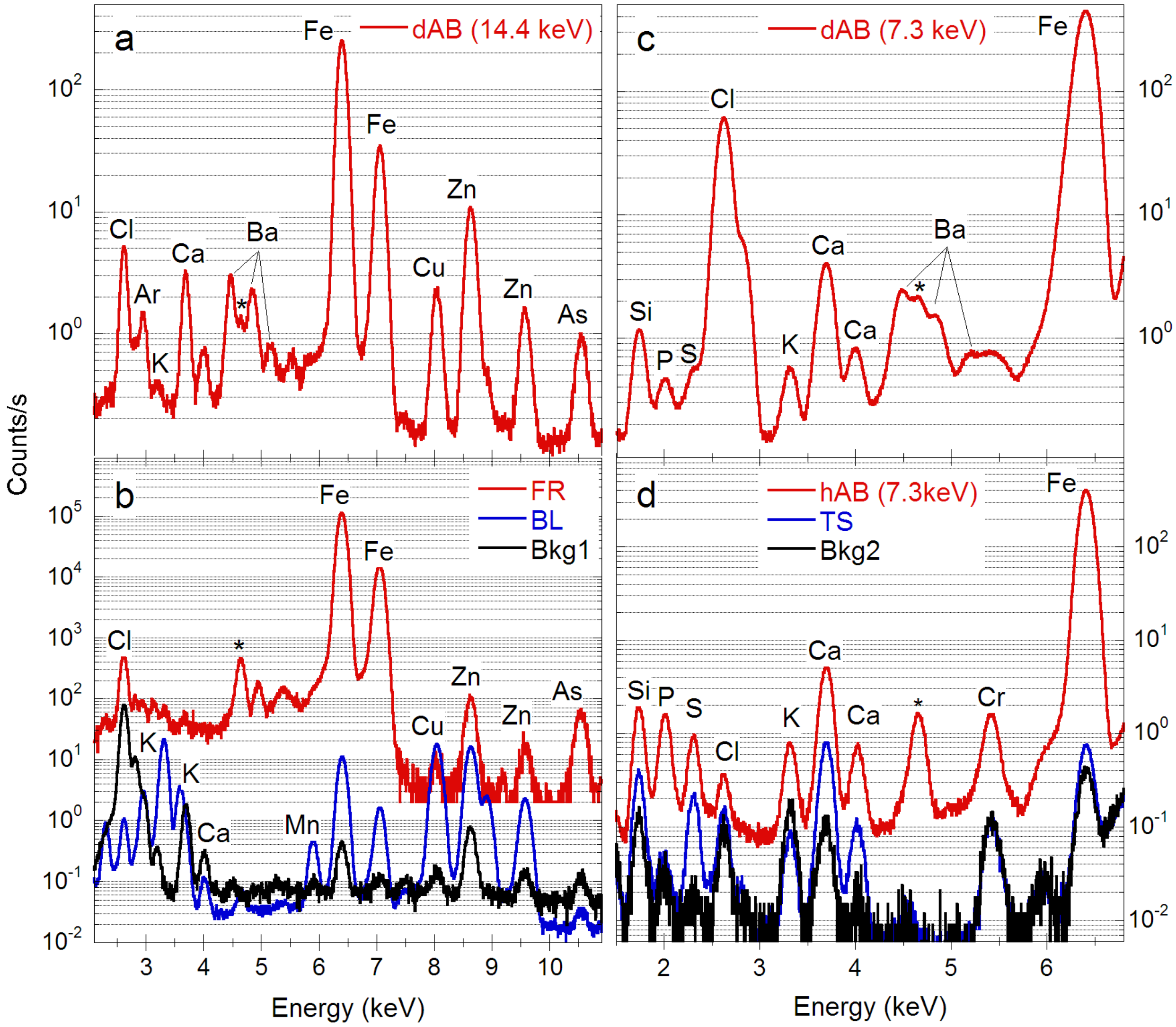

Figure S3. Representative x-ray fluorescence spectra of maps acquired at 14.4 keV, in air atmosphere at the ID18F beamline (a, b), and at 7.3 keV in vacuum at the ID21 beamline (c, d). (a) Integrated fluorescence spectrum of a map with a d*AB* acquired at 14.4 keV (b) Integrated fluorescence spectra of the ferritin (FR) and bovine liver (BL) standards, and of a porous membrane filtered with the same commercial NaClO solution used to digest the lung tissue (Bkg1) acquired at 14.4 keV. (c) Integrated fluorescence spectrum of map with a d*AB* acquired at 7.3 keV. (d) Integrated fluorescence spectrum of a map with a h*AB* acquired at 7.3 keV, of areas of the membrane without *AB*, representing the original lung tissue (TS), and of an empty PEN membrane (polyethylene naphthalate, purchased from MMI) used to support the histological sections with an Ultralene® layer superimposed (Bkg2); significant impurities of Si, P, Cl, Ca, and Cr were present on the PEN membrane. The asterisks indicate the Si escape peak excited by the fluorescence Fe. The Ar peak in panels (a) and (b) is due to atmospheric Ar_2 gas. The presence of Cl in the spectra of the d*AB* (panels a and c) is due to residual NaCl crystals formed during digestion of the lung tissue with NaClO. Not all peaks were labelled for clarity. The y-axes are in logarithmic scale. The dwell time was 1s/pixel for the maps acquired at 14.4 keV, and 200ms/pixel for the maps acquired at 7.3 keV.

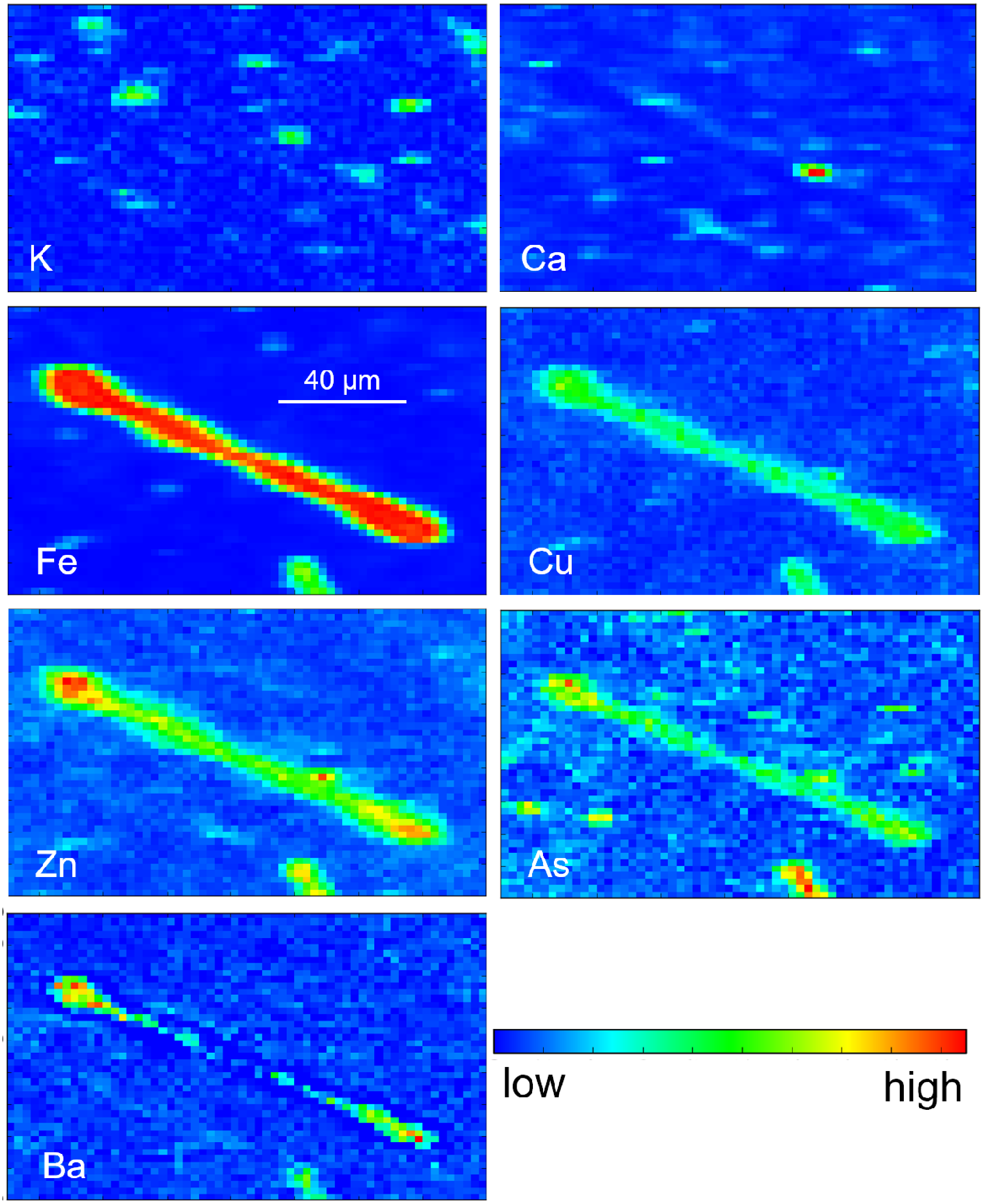

Figure S4. µXRF distribution maps of a d*AB* (case A) acquired in air at ID18F at 14.4 keV. The pixel size is 2.5 x 2.0 μm^2. The background signal (outside the *AB* area) is due to residual lung tissue deposited on cellulose esters membrane (Millipore).

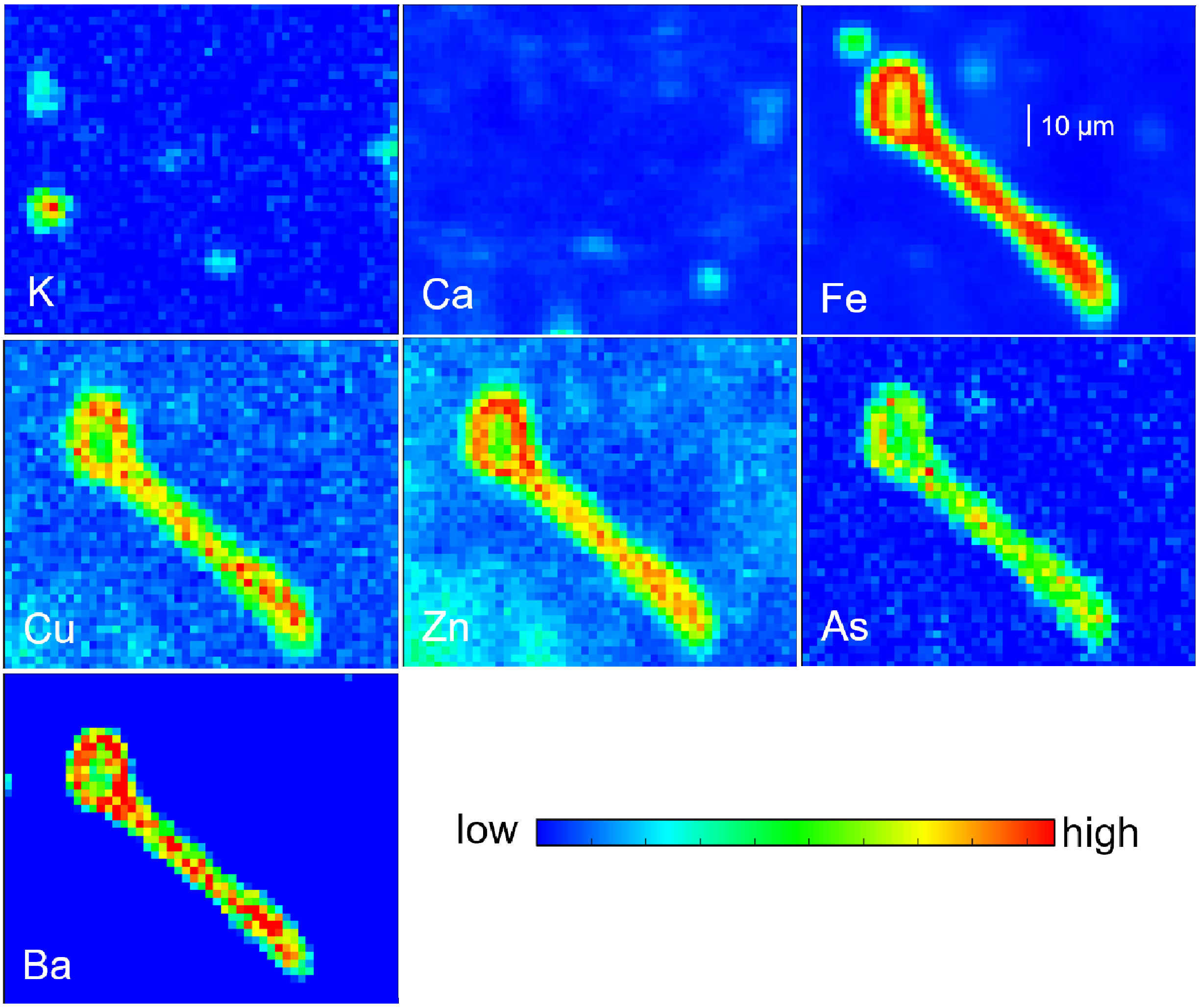

Figure S5. µXRF distribution maps of a d*AB* (case B) acquired in air at ID18F at 14.4 keV. The pixel size is 2.5 x 2.0 µm^2. The background signal (outside the *AB* area) is due to residual lung tissue deposited on cellulose esters membrane (Millipore).

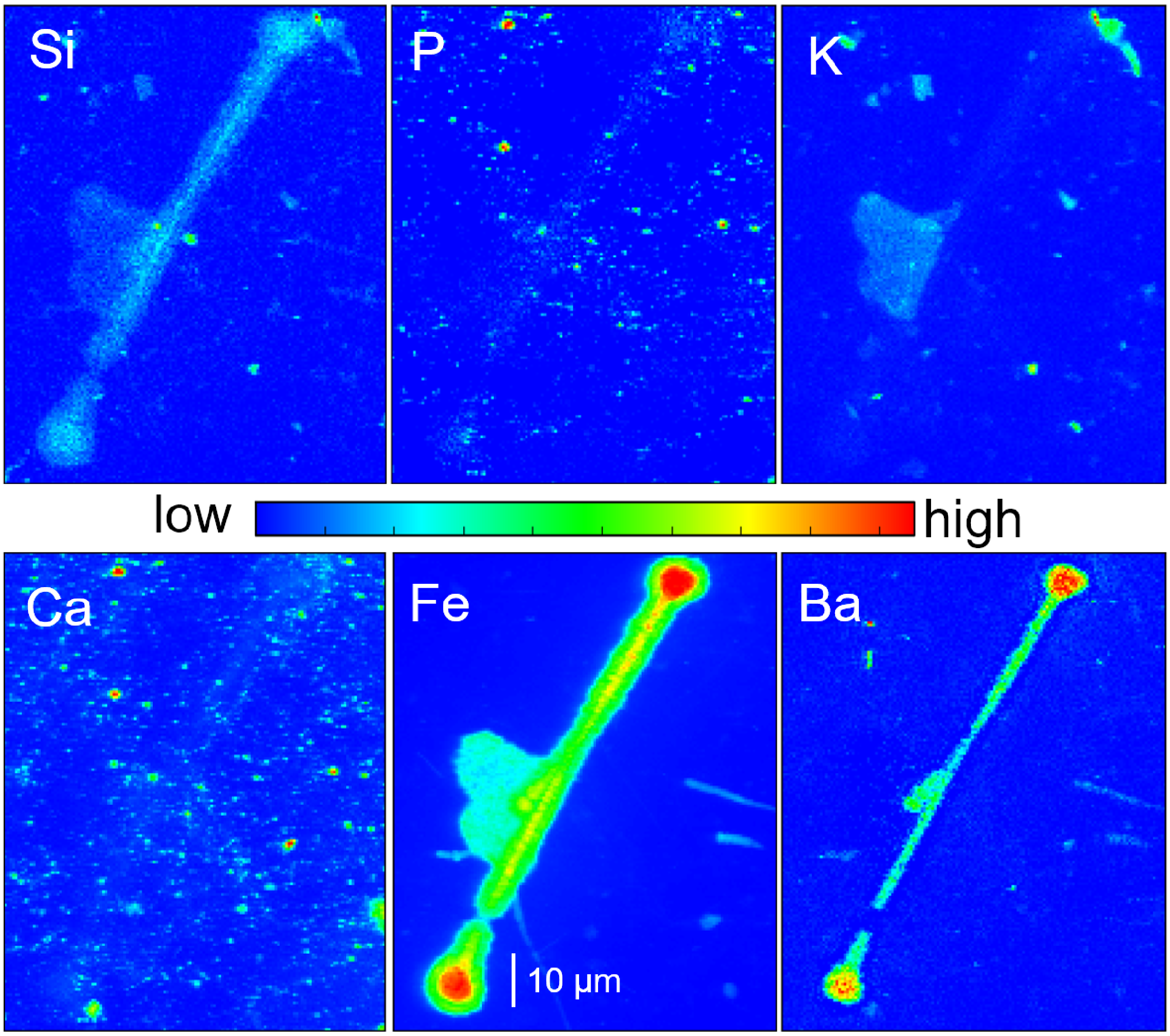

Figure S6. µXRF distribution maps of a d*AB* (case A) acquired in vacuum at ID21 at 7.3 keV. The pixel size is 0.5 x 0.5 µm^2. The background signal (outside the *AB* area) is due to residual lung tissue deposited on cellulose esters membrane (Millipore).

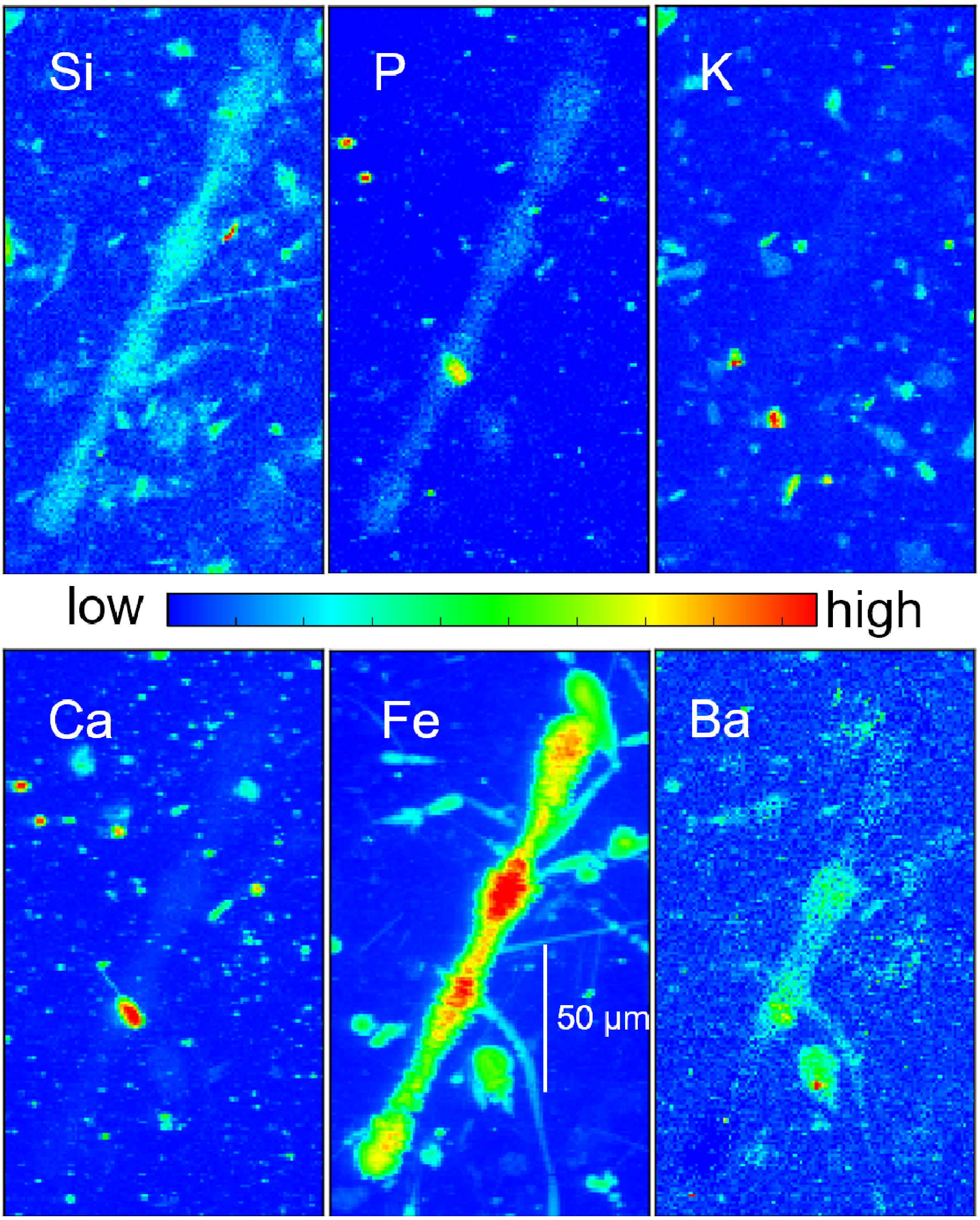

Figure S7. µXRF distribution maps of a d*AB* (case B) acquired in vacuum at ID21 at 7.3 keV. The pixel size is 0.5 x 0.5 µm^2. The background signal (outside the *AB* area) is due to residual lung tissue deposited on cellulose esters membrane (Millipore).

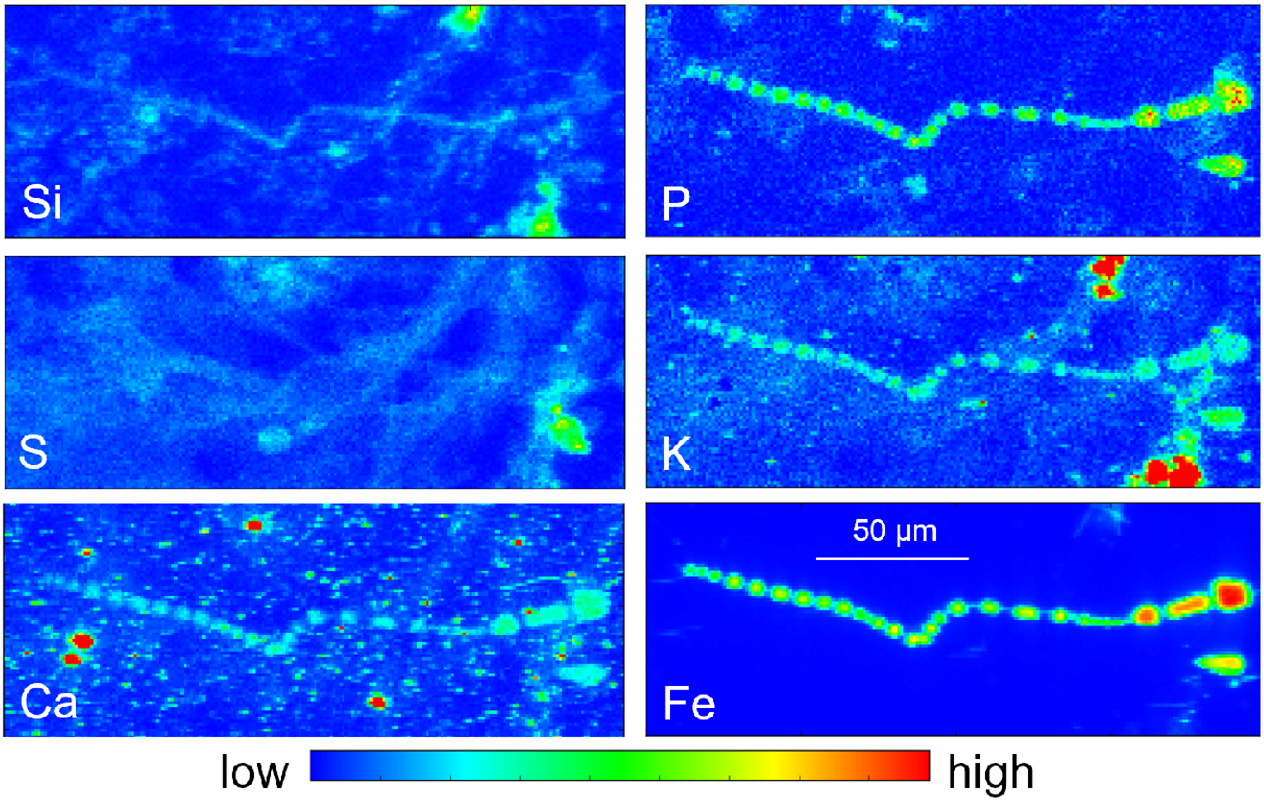

Figure S8. µXRF distribution maps of a h*AB* (case A) acquired in vacuum at ID21 at 7.3 keV. The pixel size is 0.5 x 0.5 µm^2. The background signal (outside the *AB* area) is the lung tissue supported on a PEN membrane (MMI) covered with 4 µm of Ultralene® film. Si, P, K and Ca impurities were present on the PEN membrane (see Bkg2 signal in Figure S3).

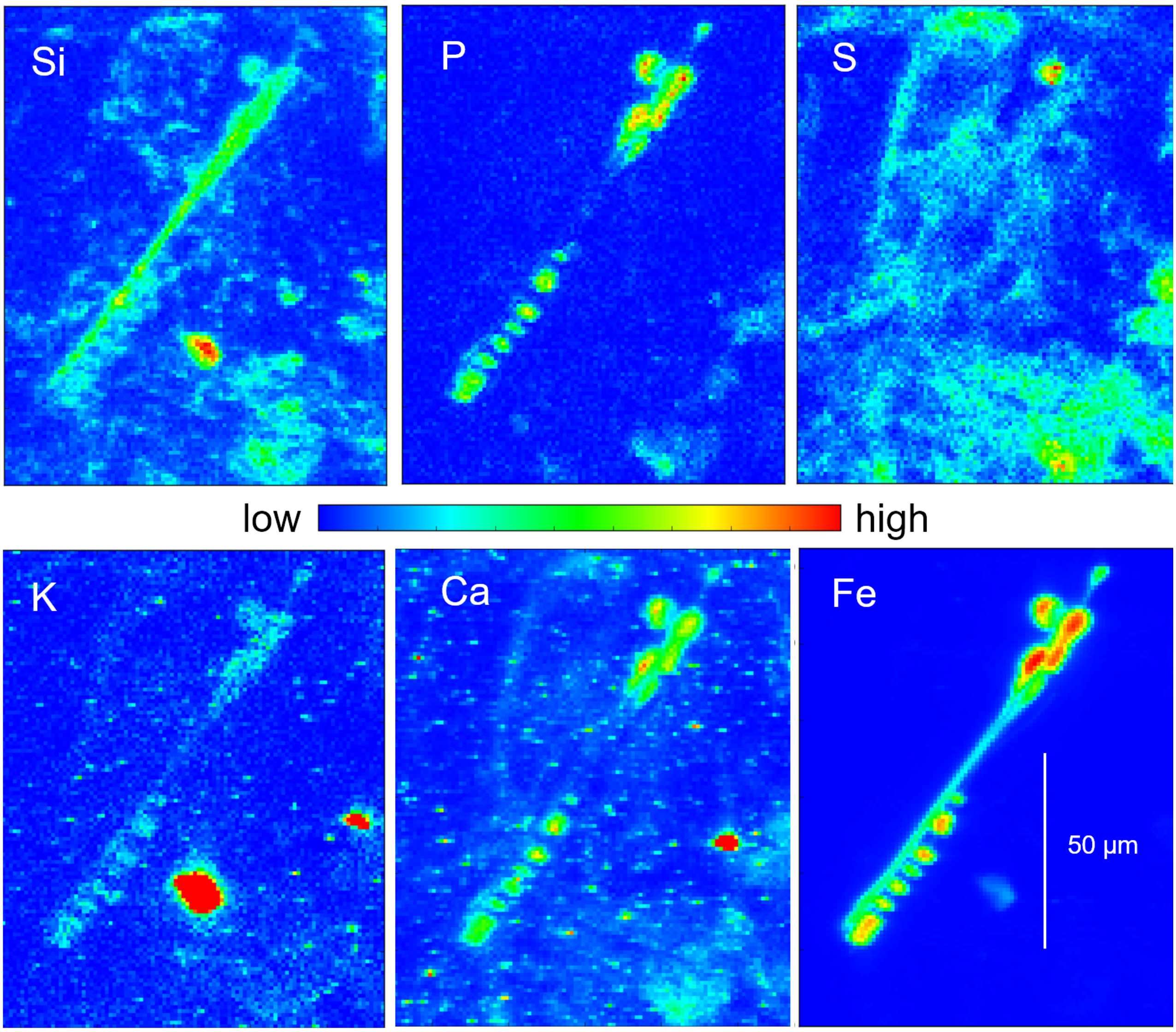

Figure S9. µXRF distribution maps of a h*AB* (case A) acquired in vacuum at ID21 at 7.3 keV. The pixel size is 0.5 x 0.5 µm^2. The background signal (outside the *AB* area) is the lung tissue supported on a PEN membrane (MMI) covered with 4 µm of Ultralene® film. Si, P, K and Ca impurities are present on the PEN membrane (see Bkg2 signal in Figure S3).

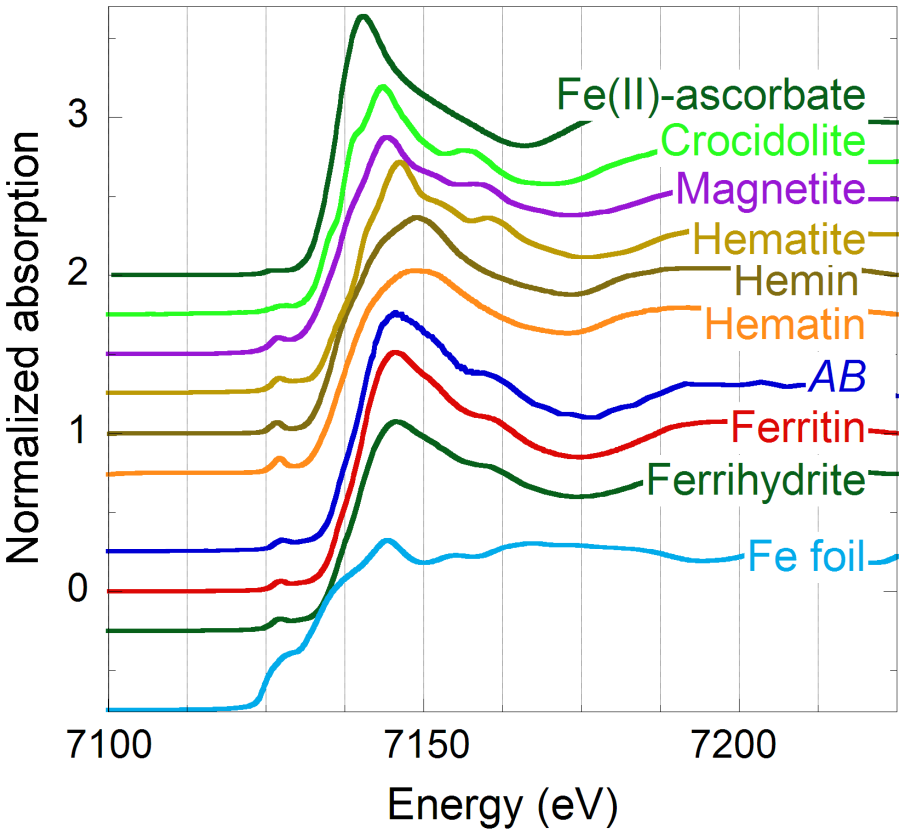

Figure S10. Background subtracted and normalized µXANES spectra of a h*AB* (*AB*) acquired at ID21 in fluorescence mode and of relevant reference Fe-compounds. All spectra were energy calibrated using the spectrum of a Fe foil acquired during the same experimental campaign.

Supporting information to the Methods section

Table S3. Summary table of the samples/experimental conditions of the synchrotron radiation experiments (RT = Room Temperature)

Samples	Maps [a]	Beamline	Source	Sample environment	Techniques	Energy (keV)	Beam size H x V (μm^2)
d*AB*/h*AB*	4/8	ID21	ESRF [b]	vacuum - RT	µXRF – µXAS	7.3	0.5 x 0.5
d*AB*	9	ID18F	ESRF [b]	Air - RT	µXRF – µXRD [c]	14.4	2.5 x 2.0

[a] Number of acquired XRF maps including an *AB;*

[b] European Synchrotron Radiation Facility (Grenoble, France);

[c] µXRD measurements were inconclusive due to the low scattering signal from single *AB*.

Synchrotron radiation experiments. The beamlines were the experiments were conducted exploit synchrotron radiation generated by in-vacuum undulators and fixed exit Si(111) double crystal monochromators to deliver a high brilliance and tuneable monochromatic x-ray beam. The focusing elements at ID18F consisted in a set of compound refractive lenses able to focus the x-ray beam down to 2.5x2.0 μm^2. The intensity of the focused beam is typically $5{\cdot}10^{10}$ photons/s. A solid state Si(Li) detector (Gresham) of 30 mm^2 active area and 3.5 µm active thickness was used to acquire fluorescence maps at a fixed energy of 14.4 keV. The ID21 beamline is equipped with a scanning x-ray microscope optimized for micro x-ray fluorescence (µXRF) imaging and micro x-ray absorption spectroscopy (µXAS). A zone plates focusing system allows for a sub-micrometric lateral resolution (0.5 x 0.5 μm^2) reaching an intensity of about 10^{10} photons/s at 7keV. The microscope is operated under high vacuum (~10^{-5} mbar) to allow the detection of low-Z elements. Fluorescence maps were acquired using an 80mm^2 active area silicon drift detector (Bruker) at 7.3 keV.

Elemental quantification. Matrix absorption corrections was applied by carefully defining a matrix as close as possible to the actual one. In the case of the 10µm-thick histological sections measured at ID21, the matrix was defined as a ferruginous material (i.e. the *AB*) of few µm thickness between two 5µm-thick layers of biological tissue, which roughly correspond to the total thickness of the histological sections. The *AB* was modelled starting from the results of EDS quantification that indicated a Fe level on the *AB* in range between 15 and 30% wt. (average value 27% wt.), and that of x-ray absorption spectroscopy that indicated that Fe in the *AB* mainly occurs in the form of ferritin, which contain about 20% ferrihydrite. The amount of Fe was then adjusted *a posteriori* from the results of the XRF quantification and iteratively checked for consistency. Starting from these assumptions, the density of the *AB* layer in the absorbing matrix was set to the weighted average between that of ferritin (approximated to the protein density reported in Quillin *et al.* (2000)[2], i.e.

1.2 g/cm^3) and that of ferrihydrite (3.5 g/cm^3) [3]. The final density (1.7 g/cm^3) was then calculated assuming that ferritin was loaded with 20% Fe in the form of ferrihydrite. The composition of the tissue layers was defined as the composition of the standard biological tissue reported by NIST. XRF quantification of the bovine liver certified standard from NIST resulted in similar composition, confirming the correctness of the quantification performed. Both d*AB* deposited on porous membranes and h*AB* supported on PEN membranes were covered by a layer of 4µm-thick polymer film (Ultralene®) whose effect was considered in the quantification procedure. The main sources of uncertainty in the determination of the elemental concentration come from the error in the calculation of the *AB* thickness and density. The average thickness of each *AB* was determined from the corresponding XRF maps. Nevertheless, due to their limited lateral resolution and to the irregular shape of the *AB*, the thickness can be under or overestimated by up to one pixel (2–2.5µm), which corresponds to an error of up to 40-50% on the concentrations. In particular, due to the limited resolution, the *AB* diameter calculated from SEM images was always lower than that calculated from XRF maps. For this reason the pixels located at the *AB's* borders in the XRF maps were neglected, and the thickness was usually estimated in the narrower part of the *AB*. On the other hand, wrong density would account to up to 50% error on the concentrations, has observed choosing one of the two end densities (i.e. apoferritin or pure ferrihydrite). Considering the above points, the absolute error on the concentrations was estimated to be lower than 50% of the values reported.

Elemental quantification was performed using the PyMCA software package[4], which allows the deconvolution of the fluorescence signal of each element. Silicon escape peaks from Fe fluorescence (Figure S1) and sum up peaks from Fe, when present, accounted for 0.3-0.4% of the parent peaks. The scattering peak was included in the fit of the fluorescence spectra. The absorbing matrix (defined as described above), the absorbers between the sample and the detector (air, Kapton, Ultralene® and Be windows, detector active and dead Si layers), and the fundamental parameters (photon flux, integration time, sample to detector distance, and detector active area) were set. The photon flux was adjusted using the certified standards (see main text). The sensitivity was estimated to be of 0.001% w/w down to the Kα fluorescence line of K.

X-ray Absorption Spectroscopy. Linear Combination Fitting (LCF) consists in a weighted linear combination of the reference standards to reconstruct a given experimental spectrum. Provided that the set of reference compounds is sufficiently representative, LCF can reveal the relative amounts of the main species present in the samples. Starting from the best two components fit, the best fit with n + 1 components was considered to be significantly better than the best n-component one, if the reduced square fit (χ_ν^2) was at least 15% lower than that of the best n-component fit[5]. The error on the values obtained following this method was previously estimated to be between 10 and 15%[5,6], and strongly depends on the signal to noise ratio and on possible errors in the calibration and normalization of the spectra. Fractions present in amounts <10% were neglected since these amounts were comparable to the error associated with this technique and because

they were found to improve the fits negligibly. LCF was performed using with the IFEFFIT code (Ravel and Newville, 2005).

The oxidation state of Fe was determined following the work of Wilke *et al.* (2001)[7], who demonstrated that the relevant parameter to determine the oxidation state of iron from a XANES spectrum is not the energy position of the absorption edge, which depends strongly on iron speciation, but the position of the centroid of the pre-edge peaks, which are related to 1s–3d or 4d electronic transitions[7]. After calibration and normalization of the XANES spectra, an edge-shaped baseline was subtracted from the absorption spectra, and pre-edge peaks were fitted using pseudo-Voigt or Gaussian functions. The position of the centroid was then determined by averaging the energy positions of the peaks weighted by their area.

References

1. Hawthorne, F. C. & Oberti, R. Classification of the Amphiboles. *Rev. Mineral. Geochemistry* **67,** 55–88 (2007).
2. Quillin, M. L. & Matthews, B. W. Accurate calculation of the density of proteins. *Acta Crystallogr. D. Biol. Crystallogr.* **56,** 791–4 (2000).
3. Hiemstra, T. & Van Riemsdijk, W. H. A surface structural model for ferrihydrite I: Sites related to primary charge, molar mass, and mass density. *Geochim. Cosmochim. Acta* **73,** 4423–4436 (2009).
4. Solé, V. A., Papillon, E., Cotte, M., Walter, P. & Susini, J. A multiplatform code for the analysis of energy-dispersive X-ray fluorescence spectra. *Spectrochim. Acta Part B At. Spectrosc.* **62,** 63–68 (2007).
5. Isaure, M.-P. *et al.* Quantitative Zn speciation in a contaminated dredged sediment by µ-PIXE, µ-SXRF, EXAFS spectroscopy and principal component analysis. *Geochim. Cosmochim. Acta* **66,** 1549–1567 (2002).
6. Bardelli, F., Cattaruzza, E., Gonella, F., Rampazzo, G. & Valotto, G. Fe and Mn speciation in road dust particles by XAS. *J. Phys. Conf. Ser.* **190,** 12192 (2009).
7. Wilke, M., Farges, F., Petit, P. E., Brown, G. E. & Martin, F. Oxidation state and coordination of Fe in minerals: An FeK- XANES spectroscopic study. *Am. Mineral.* **86,** 714–730 (2001).
